# Aging-Related Decline of Autophagy in Patients with Atrial Fibrillation—A Post Hoc Analysis of the ATHERO-AF Study

**DOI:** 10.3390/antiox11040698

**Published:** 2022-04-01

**Authors:** Francesco Versaci, Valentina Valenti, Maurizio Forte, Vittoria Cammisotto, Cristina Nocella, Simona Bartimoccia, Leonardo Schirone, Sonia Schiavon, Daniele Vecchio, Luca D’Ambrosio, Giulia Spinosa, Alessandra D’Amico, Isotta Chimenti, Francesco Violi, Giacomo Frati, Pasquale Pignatelli, Sebastiano Sciarretta, Daniele Pastori, Roberto Carnevale

**Affiliations:** 1 Department of Cardiology, Santa Maria Goretti Hospital, 04100 Latina, Italy; francescoversaci@yahoo.it (F.V.); valevale2012@hotmail.com (V.V.); 2 IRCCS Neuromed, 86077 Pozzilli, Italy; maur.forte@gmail.com (M.F.); fraticello@inwind.it (G.F.); sebastiano.sciarretta@uniroma1.it (S.S.); 3 Department of Clinical Internal, Anesthesiological and Cardiovascular Sciences, Sapienza University of Rome, 00161 Rome, Italy; vittoria.cammisotto@uniroma1.it (V.C.); cristina.nocella@uniroma1.it (C.N.); pasquale.pignatelli@uniroma1.it (P.P.); 4 Department of Medical-Surgical Sciences and Biotechnologies, Sapienza University of Rome, 04100 Latina, Italy; simona.bartimoccia@uniroma1.it (S.B.); leonardo.schirone@uniroma1.it (L.S.); sonia.schiavon@uniroma1.it (S.S.); danielevecchio888@gmail.com (D.V.); l.dambrosio@uniroma1.it (L.D.); spinosa.1690694@studenti.uniroma1.it (G.S.); isotta.chimenti@uniroma1.it (I.C.); 5 Department of Movement, Human and Health Sciences, University of Rome “Foro Italico”, 00135 Rome, Italy; a.damico@studenti.uniroma4.it; 6 Mediterranea Cardiocentro, 80122 Naples, Italy; francesco.violi@uniroma1.it

**Keywords:** atrial fibrillation, aging, autophagy, cardiovascular disease, oxidative stress

## Abstract

Background: Aging is an independent risk factor for cardiovascular diseases. The autophagy process may play a role in delaying aging and improving cardiovascular function in aging. Data regarding autophagy in atrial fibrillation (AF) patients are lacking. Methods: A post hoc analysis of the prospective ATHERO-AF cohort study, including 150 AF patients and 150 sex- and age-matched control subjects (CS), was performed. For the analysis, the population was divided into three age groups: <50–60, 61–70, and >70 years. Oxidative stress (Nox2 activity and hydrogen peroxide, H_2_O_2_), platelet activation (PA) by sP-selectin and CD40L, endothelial dysfunction (nitric oxide, NO), and autophagy parameters (P62 and ATG5 levels) were assessed. Results: Nox2 activity and H_2_O_2_ production were higher in the AF patients than in the CS; conversely, antioxidant capacity was decreased in the AF patients compared to the CS, as was NO production. Moreover, sP-selectin and CD40L were higher in the AF patients than in the CS. The autophagy process was also significantly impaired in the AF patients. We found a significant difference in oxidative stress, PA, NO production, and autophagy across the age groups. Autophagy markers correlated with oxidative stress, PA, and endothelial dysfunction in both groups. Conclusions: This study provides evidence that the autophagy process may represent a mechanism for increased cardiovascular risk in the AF population.

## 1. Introduction

The increase in life expectancy observed in recent decades, thanks to advances in medical therapies or the improvement of lifestyles, is unfortunately associated with the higher incidence of diseases related to aging, especially cardiovascular diseases (CVDs) [1,2]. Risk factors associated with CVDs, such as hypertension and atherosclerosis, generally increase in the elderly population, while they are rare in the young population [3,4]. There are several mechanisms of aging, including progressive oxidative damage and increased age-related platelet activation [5]. A deeper understanding of the molecular mechanisms involved in the aging of the cardiovascular system may facilitate targeted therapies able to retard cardiovascular aging. Preclinical evidence suggests that autophagy is one of the main processes altered during aging [6]. Autophagy is an intracellular catabolic recycling process that removes senescent or damaged cytoplasmic elements, including organelles. It consists of the formation of double-membrane vesicles called autophagosomes, which sequester cytoplasmic cargoes that are then delivered and digested into lysosomes [7]. Autophagy ensures cardiovascular homeostasis in unstressed and stressed conditions [8,9]. During aging, autophagy decreases, leading to harmful cardiovascular effects, since toxic products that generally accumulate in aged districts are not adequately removed and recycled [6,8]. The latter also contributes to increased oxidative stress, with consequent damage to proteins and DNA, organelle dysfunction, and cell death. Therapeutic strategies capable of increasing autophagy, such as caloric restriction as well as synthetic or natural products, have been shown to prolong lifespans and improve cardiovascular function in different experimental models of aging [10,11]. These data suggest that improving autophagy may be an interesting approach to delaying aging-related diseases. In recent years, circulating levels of autophagic markers are also emerging as valid diagnostic/prognostic tools in various human diseases, such as ischemic stroke and Alzheimer’s disease [12,13]. Atrial fibrillation (AF) is the most common cardiac arrhythmia and represents an aging-related condition [14]. Oxidative stress and inflammation represent the main pathogenic determinants that contribute to AF onset and maintenance. Patients suffering from AF also show an increase in platelet aggregation and vascular dysfunction [15,16]. For this reason, AF is considered as one of the main risk factors for the development of adverse cardiovascular events, such as myocardial infarction and ischemic stroke [17,18]. Of note in AF, impaired oxidative status and increased platelet activation seem to be age-correlated [19,20]. However, the role of autophagy in patients with AF has not been well-established. In this prospective cohort study, we investigated whether the level of autophagy correlates with aging as well as with markers of oxidative stress, NO production, and platelet reactivity in control individuals and patients with AF.

## 2. Materials and Methods

### 2.1. Population Study

We performed a post hoc analysis of 150 nonvalvular AF patients included in the prospective ongoing ATHERO-AF cohort study, and 150 control subjects (CS). Patients were randomly selected from the original cohort. Patients and CS were balanced for age and sex and divided into three age groups: (1) <50–60 years, (2) 61–70 years, and (3) >70 years. AF patients were recruited from the Atherothrombosis Centre of the Department of Clinical, Internal, Anesthesiological and Cardiovascular Sciences, of the Sapienza University of Rome, for the monitoring and management of antithrombotic therapies. All patients were treated with vitamin K antagonists (warfarin), and none of patients were being treated with antiplatelet drugs. None of the CS were being treated with vitamin K antagonists (warfarin).

All patients provided written informed consent at baseline. The study protocol was approved by the local ethical board of the Sapienza University of Rome (ethical protocol code is 1306/2007) and was conducted according to principles of the Declaration of Helsinki. The study is registered at clinicaltrials.gov accessed on 26 March 2022, NCT01882114.

### 2.2. Preparation of Serum, Plasma, and Platelets

Plasma and serum samples were collected in BD Vacutainers (Franklin Lakes, NJ, USA) with or without anticoagulant (trisodium citrate, 3.8%, 1/10 (*v*:*v*)), respectively. The blood was centrifuged at 300 g for 10 min at room temperature (RT). The supernatants were divided into aliquots and stored at −80 °C for analyses. The study protocol was approved by the local ethical board of the Sapienza University of Rome and conducted according to principles of the Declaration of Helsinki.

### 2.3. Serum sNox2-dp Release

Nox2 activation was determined as soluble Nox2-derived peptide (sNox2-dp) with an ELISA method as previously reported [21]. In brief, the peptide is recognized by binding to a specific monoclonal antibody against the amino acid sequence (224–268), extra-membrane domain of Nox2, which was released after platelet activation. The enzyme activity is measured spectrophotometrically by the increased absorbency at 450 nm. Values were expressed as pg/mL; intra-assay and interassay coefficients of variation were 8.95% and 9.01%, respectively.

### 2.4. Serum H_2_O_2_ Determination

Hydrogen peroxide (H_2_O_2_) in serum was evaluated by a Colorimetric Detection Kit (Arbor Assays, Ann Arbor, MI, USA). Values were expressed as μM. Intra-assay and interassay coefficients of variation were 2.1% and 3.7%, respectively.

### 2.5. Serum Hydrogen Peroxide Scavenging Activity

To assess the antioxidant capacity, we measured serum hydrogen peroxide (H_2_O_2_) breakdown activity (HBA) by an HBA assay kit (Aurogene, Roma, Italy, code HPSA-50). The % of HBA was calculated according to the following formula: % of HBA = [(Ac − As)/Ac] × 100, where Ac is the absorbance of H_2_O_2_ 1.4 mg/mL and As is the absorbance in the presence of the serum sample.

### 2.6. Serum Nitric Oxide

For the measurement of nitric oxide (NO) in serum, we used a colorimetric assay kit (Cell Biolabs, San Diego, CA, USA) that quantitatively measures NO by NO^2−^/NO^3−^ determination. Briefly the nitrate (NO^3−^) in the sample is initially converted into nitrite (NO^2−^) by nitrate reductase enzyme; next, total nitrite is detected with Griess reagents as a colored azo dye product (absorbance: 540 nm). Values were expressed as μM. Intra- and interassay coefficients of variation were <10%.

### 2.7. Plasma sP-Selectin Assay

P-selectin is an adhesion molecule stored in platelet α-granules and released upon activation as a soluble form. Platelets are the main sources of circulating sP-selectin, which is a reliable marker of platelet activation [22].

Plasma sP-selectin levels were evaluated by a commercial immunoassay (Diaclone), and values are expressed as ng/mL; intra- and interassay coefficients of variation were 5.6% and 7.5%, respectively.

### 2.8. Plasma CD40L Assay

CD40L is a member of the tumor necrosis factor (TNF) family, stored in α-granules and released after activation. Platelets are a significant reservoir of CD40L, which is a molecular driver of platelet-induced processes such as inflammation, coagulation, tissue remodeling, and host defense [23].

Plasma levels of soluble CD40 ligand (sCD40L) were measured with a commercial immunoassay (Quantikine CD40 ligand, R&D Systems Inc., Minneapolis, MN, USA). Intra-assay and interassay coefficients of variation were 7% and 9%, respectively.

### 2.9. Plasmatic ATG5 Detection

ATG5 belongs to the ATG family. ATG5 plays an essential role in the process of autophagy for its role in the early stages of autophagosome formation, a double-membrane vesicle that sequesters cytoplasmic material before lysosomal delivery [24].

For the quantitative determination of autophagy protein 5 (ATG5) concentrations in plasma samples, we used the sandwich enzyme immunoassay technique (Mybiosource, San Diego, CA, USA, No. MBS2602759). The sample concentrations were determined using a microplate reader set to 450 nm, and values were expressed as ng/mL. Intra-assay and interassay coefficients of variation were ≤8% and ≤12%, respectively.

### 2.10. Plasmatic P62 Detection

P62 is a ubiquitin-binding scaffold protein that may serve to deliver ubiquitinated substrates to the autophagic machinery to enable their degradation in the lysosome. Since P62 is itself degraded by autophagy and accumulates when autophagy is inhibited, it may be used as a marker of autophagic flux [25].

Plasmatic P62 was analyzed with sandwich enzyme immunoassay technology (FineTest, Wuhan, China, No. EH10842). The concentration of protein can be calculated by reading the O.D. absorbance at 450 nm. Values were expressed as ng/mL. Intra-assay and interassay coefficients of variation were <8 and <10%, respectively.

### 2.11. Statistical Analysis

Categorical variables were reported as counts/percentages and continuous variables as means (± standard deviation). A Student’s unpaired t-test was used to compare means, and a two-way ANOVA was used to compare groups with a post hoc LSD test. Spearman correlation analysis and univariable linear regression analysis were used to assess the association between markers of autophagy and oxidative stress, platelet activation, and endothelial dysfunction parameters. *p*-values < 0.05 were considered as statistically significant. All tests were two-tailed, and analyses were performed using GraphPad Prism9.1.0 and IBM SPSS 25.03.

## 3. Results

### 3.1. Characteristics of Population

Clinical and demographic characteristics are summarized in Table 1.

### 3.2. Oxidative Stress Analysis

Overall, the AF patients presented higher levels of sNox2-dp and H_2_O_2_ production as well as lower antioxidant capacity evaluated by HBA compared to the CS (Table 2).

**Figure 1:**
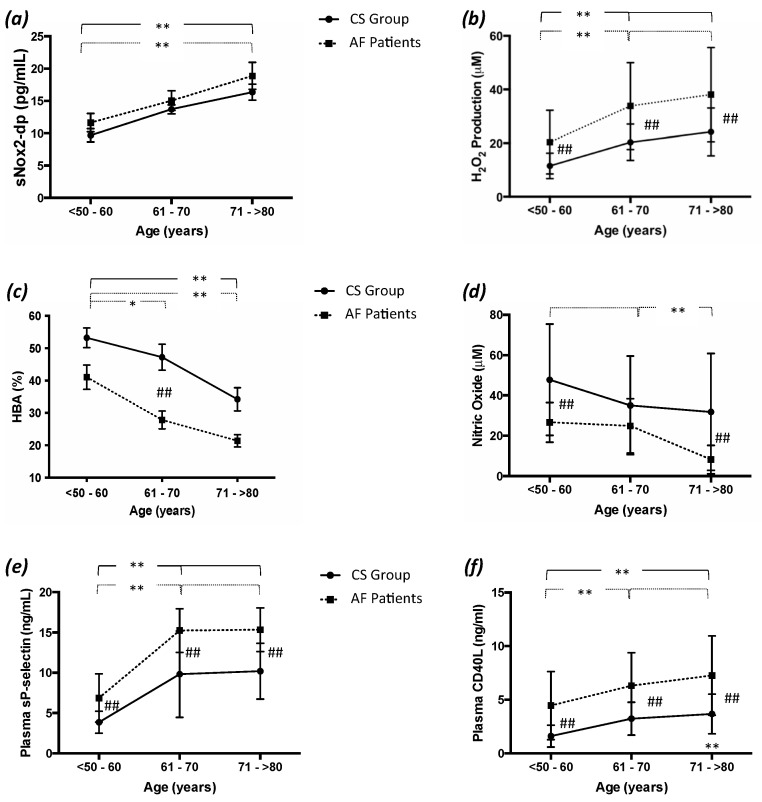
Oxidative stress, endothelial dysfunction, and platelet activation in aging. (**a**) Serum sNox2-dp release; (**b**) serum hydrogen peroxide (H_2_O_2_) production; (**c**) serum H_2_O_2_ breakdown activity (HBA); (**d**) serum nitic oxide (NO); (**e**) plasma sP-selectin; and (**f**) plasma CD40L in control subjects (CS, n = 150) and atrial fibrillation patients (AF, n = 150) divided into three age groups (<50–60; 61–70; and >70 years). Data are expressed as median and SD. Intragroup significance: * *p* < 0.05, ** *p* < 0.01; intergroup significance: ## *p* < 0.01.

To evaluate the impact of age on the AF patients and the CS group, we divided our population into three different age classes; we found a significant difference in oxidative stress parameters across these classes (Figure 1a–c). In particular, both the AF patients and the CS in the third age class showed increased sNox2-dp release compared to the first age class. No change was observed between the AF patients and the CS for all age classes (Figure 1a).

H_2_O_2_ production was significantly higher in the second and third age classes of the AF patients and the CS compared to the first one (Figure 1b). Significant differences were observed between the AF patients and the CS in the three age classes (Figure 1b). The antioxidant activity was lower in the third and second age classes of the AF patients compared to the first (Figure 1c). However, we found significant differences in the CS only if we compared the third age class with the first. Furthermore, significant differences were observed between the AF patients and the CS in the second age class (Figure 1c).

### 3.3. NO Production Analysis

As shown in Table 2, the AF patients displayed worse serum NO production. Indeed, the levels of NO in the AF patients were significantly lower than in the CS (Table 2). Furthermore, serum NO was significantly decreased in the AF patients included in the third age class compared to the second and first classes (Figure 1d), whereas no change was found in the CS subjects across classes. Moreover, significant differences were observed between the AF patients and the CS in the first and third age classes (Figure 1d).

### 3.4. Platelet Activation Analysis

The analysis of platelet activation markers, such as sP-selectin and CD40L, relieved significantly differences between AF patients and CS (Table 2). In addition, we observed that platelet activation was significantly higher in AF patients and CS specially in the oldest population. In fact, the levels of sP-selectin and CD40L were significantly greater in the second and third age classes than in the first (Figure 1e,f). Moreover, no changes were observed between the second and first age classes for CD40L levels (Figure 1e,f). Finally, significant differences were observed between the AF patients and the CS in all age classes (Figure 1e,f).

### 3.5. Autophagy Process Analysis

**Figure 2:**
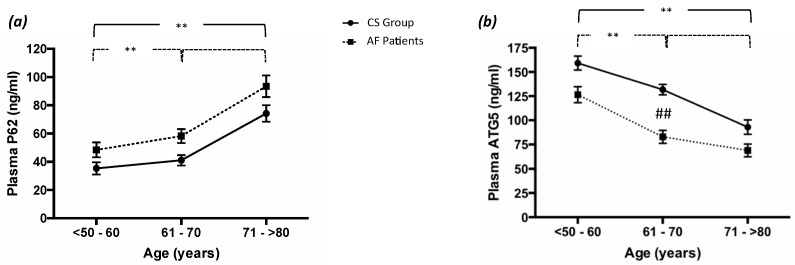
Autophagy process and aging. (**a**) Plasma p62 and (**b**) ATG5 levels in in control subjects (CS, n = 150) and atrial fibrillation patients (AF, n = 150) divided into three age groups (<50–60 years; 61–70 years; and >70 years). Data are expressed as median and SD. Intragroup significance: ** *p* < 0.01; intergroup significance: ## *p* < 0.01.

To explore the role of autophagy, we evaluated the levels of P62 and ATG5 in the plasma of the AF patients and the CS. This analysis revealed that, compared to the CS, the AF patients showed an increase in P62 levels and a reduction in ATG5 levels (Table 2). Moreover, the AF patients showed a significant decrease in the autophagic process across age classes (Figure 2a,b). In particular, the P62 levels were significantly augmented in subjects included in the third age class compared to those in the second and first age classes (Figure 2a). Conversely, levels of the ATG5 protein were significantly reduced in subjects of the second and third age classes compared to those of the first (Figure 2b). Instead, the CS only showed significant differences in both P62 and ATG5 levels between the third age class compared to the first (Figure 2 a,b). No significantly differences were detected between groups for P62 levels (Figure 2a). Instead, we observed significant differences between the two groups for ATG5 levels for the second age class as well (Figure 2b).

### 3.6. Correlation Analysis in the AF Patients and CS

Correlation analysis showed significant correlations for both the AF patients and the CS between markers of autophagy and oxidative stress, platelet activation, and endothelial dysfunction parameters (Table 3). Correlation analysis showed that plasma P62 levels were significantly correlated with age both in the control groups and AF patients (rS = 0.138, β: 0.372, *p* < 0.001; rS = 0.080, β: 0.283, *p* < 0.001, respectively). Similarly, plasma ATG5 levels in the control groups and AF patients were also significantly correlated with age (rS = 0.229, β: −0.478, *p* < 0.001; rS = 0.136, β: −0.369, *p* < 0.001, respectively). We also found a significant correlation of the thromboembolic risk, as assessed by the CHA_2_DS_2_VASc score, with autophagy and oxidative stress markers in the AF patients (Table 3).

## 4. Discussion

In this study, we evaluated the impact of aging on markers of oxidative stress, NO production, platelet reactivity, and autophagy in AF patients and CS.

The first finding of this study consists of a worse redox status in patients with AF compared to age-balanced CS, as evident from the increase in oxidative stress and platelet activation as well as the reduction in NO production and autophagy. Secondly, we found significant differences among age classes, with a linear trend towards worsening in the older age groups, both in patients with AF and in CS. Third, our regression analysis revealed a linear correlation between autophagy level and the evaluated parameters. In addition, the levels of markers of autophagy were correlated with age in both AF patients and CS. Our data suggest that the molecular mechanisms involved in endothelial and platelet function are impaired as aging proceeds, even in control individuals, perhaps due to the imbalance of the redox state and the impairment of protective recycling processes. We and other researchers have already reported marked endothelial dysfunction as well as oxidative stress, antioxidant status decline, and platelet activation in the AF population [19,20,26]. We demonstrated that prostaglandin PGF2alpha (8-iso-PGF2α), a reliable marker of oxidative stress, and NOX2, one of the most important enzymes producing ROS, were significantly increased and independently predicted cardiovascular events in AF patients, suggesting this enzymatic pathway as a trigger of oxidative stress in the AF population. Moreover, a previous study [27] demonstrated that glutathione peroxidase, an antioxidant enzyme that catabolizes hydrogen peroxide, is predictive of cardiovascular events, suggesting that a low-antioxidant status predisposes patients to poor vascular outcomes. Indeed, we found that GPx3 progressively declines with aging in AF patients, suggesting that the reduction in natural antioxidants may be a factor predisposing the elderly population to cardiovascular complications. In further support of this hypothesis, we found also that GPx3 activity was inversely associated with the urinary excretion of thromboxane B2, a marker of in vivo platelet activation, suggesting that the relationship between GPx3 and cardiovascular events may be mediated by enhanced platelet activation. Finally, Wagner et al. add important novel insights into arrhythmogesis in ventricular myocytes. The authors showed that angiotensin II (Ang II) alters multiple, potentially proarrhythmic mechanisms, including increased peak Ca and Na currents (ICa and INa) as well as enhanced sarcoplasmic reticulum (SR) Ca spark frequency in a NOX2-dependent manner. They further showed that NOX2 differentially regulates these targets via protein kinase A (PKA) and Ca/Calmodulin kinase II (CaMKII), resulting in a dramatically increased propensity for cellular arrhythmias, to which both kinases differentially contribute [28].

Here, we have provided new evidence that autophagic markers are reduced during aging, according to data reported in preclinical studies. The observation that AF patients showed reduced autophagy as compared to control individuals may suggest a causative link between autophagy and AF progression. Other studies analyzed levels of cardiac autophagy in the AF population. A marked increase in microtubule-associated protein 1 light chain 3 (LC3) expression and autophagosome accumulation have been reported in cardiac samples from patients with chronic and persistent AF, respectively [29,30]. Autophagic flux was also impaired in the cardiac tissues of patients who developed postoperative AF after cardiac bypass surgery or mitral regurgitation [31,32]. To the best of our knowledge, our study is the first to assess the level of autophagy in plasma samples of AF patients. Our results suggest that autophagy can be considered as an early marker for risk stratification in patients with AF. The evaluation of autophagy level may also represent a predictive tool to reduce the risk of developing detrimental molecular alterations related to AF, such as oxidative stress, platelet activation, and endothelial dysfunction. Indeed, the observation of a linear correlation between autophagic markers and these parameters in healthy subjects also suggests that autophagy may be considered a mechanism for predicting AF in the general population. In accordance with this hypothesis, a recent gene study showed an increased expression of 11 autophagy-related genes (CDKN1A, CXCR4, DIRAS3, HSP90AB1, ITGA3, PRKCD, TP53INP2, DAPK2, IFNG, PTK6, and TNFSF10) in AF [33]. In addition, the analysis of circular RNA (circRNA) to analyze differentially expressed circRNAs (DECs) identified a circRNA–miRNA–mRNA regulatory network consisting of 11 DECs, 9 target miRNAs, and 410 target genes [34]. These results suggest that autophagy may be implicated in the pathogenesis of AF. The use of genetic testing in the risk stratification of the general population for determining the risk of new-onset AF in addition to classical cardiovascular risk factors and oxidative markers requires further investigation [35,36].

Preclinical evidence has shown that autophagy acts as the main antiaging system. Autophagy activation prolongs lifespans and slows down aging and age-related complications by rejuvenating cellular components [6,37]. In human studies, both resistance and aerobic exercise training have been reported to activate markers of autophagy and reduce inflammation in peripheral blood mononuclear cells (PBMCs) isolated from elderly subjects [38,39]. Since several activators of autophagy are natural products, with no negative side effects [40,41], it should be interesting to evaluate in clinical trials whether the introduction of these compounds into diets would improve cardiovascular status by reducing the impact of aging.

Our study also presents limitations that need to be reported. Since this study was conducted on AF patients not subject to cardiac surgery, we do not have human atrial tissue, so we could not assess the cellular contribution to cardiovascular disease. Moreover, we evaluated systemic levels of autophagy, as there are no specific circulating markers for the evaluation of vascular and cardiac autophagy. However, we can speculate that changes in serum levels of autophagy could reflect cardiac tissue damage.

As this is a cohort study, we cannot establish a direct cause–effect relationship between AF and impaired autophagy, which could directly promote the onset of atrial fibrillation or favor changes in platelet activation, resulting in the prothrombotic or hypercoagulable state in this arrhythmia. Furthermore, autophagy has been measured only at baseline, so we do not know if it may age over time. The relatively small sample size does not allow for the investigation of the relationship of autophagy with cardiovascular outcomes. Unfortunately, our study design does not allow a proper comparison of serum levels of autophagy markers with tissue levels. However, we found that the observed alterations in circulating autophagy markers are consistent with multiple preclinical and experimental results available in the literature, showing that autophagy is impaired in response to aging and oxidative stress in the cardiovascular system. Finally, other specific markers of the NO pathway, such as cGMP or ADMA, and other specific markers of endothelial damage should be investigated to better clarify endothelial function.

## 5. Conclusions

In conclusion, our evidence indicates that an age-dependent decline in autophagy is detectable in patients with AF. Its prognostic role requires further investigation.

## Figures and Tables

**Table 1:** Characteristics of AF patients.

Risk Factor	Control Subjects	AF Patients	*p*-Value
*Arterial hypertension*	14.0%	85.3%	<0.001
*Diabetes*	4.6%	27.3%	<0.001
*Smoking*	8.7%	12.7%	n.s.
*Heart failure*	0%	18.0%	<0.001
*Previous cardiovascular disease*	2%	28.0%	<0.001
*Previous thromboembolism*	0%	12.7%	<0.001
*Time in therapeutic range (%)*	-	67.0 (54–82.0) *	-
*Creatinine Clearance (sMDRD) ml/min*	88.9 (73.1–93.3) *	78.4 (65.6–93.3) *	n.s.
*CHA_2_DS_2_VASc score*	-	3.0 (2.0–4.0) *	-

* Median, IQR.

**Table 2:** Characteristics of the study population.

Variables	Total (n = 300)	Control Subjects (n = 150)	AF Patients (n = 150)	*p*-Value
*Age (years)*		66.6 ± 9.5	66.8 ± 9.1	0.840
*Women (%)*		39.3	37.3	0.812
*Serum sNox2-dp (pg/mL)*		12.25 ± 0.58	14.60 ± 1.0012	0.04
*Serum H_2_O_2_ (µM)*		18.68 ± 0.71	30.36 ± 1.38	<0.0001
*HBA (%)*		44.89 ± 2,14	30.10 ± 1,79	<0.0001
*Plasma CD40L (ng/mL)*		2.84 ± 0.14	6.01 ± 0.28	<0.0001
*Plasma sP-selectin (ng/mL)*		7.958 ± 0.39	12.48 ± 0.40	<0.0001
*Serum NO (µM)*		38.23 ± 2,27	19.93 ± 1.08	<0.0001
*Plasma ATG5 (ng/mL)*		128.0 ± 4.45	93.21 ± 4.60	<0.0001
*Plasma P62 (ng/mL)*		50.26 ± 3.023	66.74 ± 3.81	<0.001

AF: atrial fibrillation; CS: control subjects; and HBA: hydrogen peroxide breakdown activity.

**Table 3:** Correlation analysis.

**Control Subjects Group**						
	**H_2_O_2_ (** **µ** **M)**	**P62 (ng/mL)**	**ATG5 (ng/mL)**	**NO (** **µ** **M)**	**CD40L (ng/mL)**	**sP-Selectin (ng/mL)**
**H_2_O_2_ (** **µ** **M)**	-					
**P62 (ng/mL)**	0.174 *	-				
**ATG5 (ng/mL)**	−0.233 **	−0.207 *	-			
**NO (** **µ** **M)**	−0.144	−0.025	0.085	-		
**CD40L (ng/mL)**	0.214 **	0.230 **	−0.185 *	−0.125	-	
**sP-selectin (ng/mL)**	0.244 **	0.111	−0.271 **	−0.067	0.417 **	-
**Atrial Fibrillation Patients**						
	**H_2_O_2_ (** **µ** **M)**	**P62 (ng/mL)**	**ATG5 (ng/mL)**	**NO (** **µ** **M)**	**CD40L (ng/mL)**	**sP-Selectin (ng/mL)**
**H_2_O_2_ (** **µ** **M)**	-					
**P62 (ng/mL)**	0.126	-				
**ATG5 (ng/mL)**	−0.209 *	−0.294 **	-			
**NO** **µ** **M**	−0.205 *	−0.214 **	0.250 **	-		
**CD40L (ng/mL)**	0.080	0.234 **	−0.172 *	−0.274 **	-	
**sP-selectin (ng/mL)**	0.273 **	0.242 **	−0.347 **	−0.241 **	0.281 **	-
**CHA_2_DS_2_VASc score**	0.245 **	0.241 **	−0.118	−0.326 **	0.116	0.474 **

* The correlation is significant to 0.05 (two-tailed); ** the correlation is significant to 0.01 (two-tailed).

## Data Availability

The data presented in this study are available on request from the corresponding authors.

## Abbreviations

Atrial fibrillation (AF); autophagy protein 5 (ATG5); cardiovascular diseases (CVDs); H_2_O_2_ breakdown activity (HBA); control subjects (CS); hydrogen peroxide (H_2_O_2_); light chain 3 (LC3) nitic oxide (NO); peripheral blood mononuclear cells (PBMCs); platelet activation (PA); room temperature (RT); soluble Nox2-derived peptide (sNox2-dp).
